# A multinational consensus on dysphagia in Parkinson's disease: screening, diagnosis and prognostic value

**DOI:** 10.1007/s00415-021-10739-8

**Published:** 2021-08-21

**Authors:** Giuseppe Cosentino, Micol Avenali, Antonio Schindler, Nicole Pizzorni, Cristina Montomoli, Giovanni Abbruzzese, Angelo Antonini, Filippo Barbiera, Marco Benazzo, Eduardo Elias Benarroch, Giulia Bertino, Emanuele Cereda, Pere Clavè, Pietro Cortelli, Roberto Eleopra, Chiara Ferrari, Shaheen Hamdy, Maggie-Lee Huckabee, Leonardo Lopiano, Rosario Marchese Ragona, Stefano Masiero, Emilia Michou, Antonio Occhini, Claudio Pacchetti, Ronald F. Pfeiffer, Domenico A. Restivo, Mariangela Rondanelli, Giovanni Ruoppolo, Giorgio Sandrini, Anthony H. V. Schapira, Fabrizio Stocchi, Eduardo Tolosa, Francesca Valentino, Mauro Zamboni, Roberta Zangaglia, Mario Zappia, Cristina Tassorelli, Enrico Alfonsi

**Affiliations:** 1 Department of Brain and Behavioral Sciences, University of Pavia, Via Mondino 2, 27100 Pavia, Italy; 2 Clinical Neurophysiology Unit, IRCCS Mondino Foundation, Pavia, Italy; 3 Neurorehabilitation Unit, IRCCS Mondino Foundation, Pavia, Italy; 4 Department of Biomedical and Clinical Sciences “Luigi Sacco”, University of Milan, Milan, Italy; 5 Department of Public Health, Experimental and Forensic Medicine, Unit of Biostatistics and Clinical Epidemiology, University of Pavia, Pavia, Italy; 6 Department of Neuroscience, Rehabilitation, Ophthalmology, Genetics and Maternal Child Health, University of Genoa-IRCCS Ospedale Policlinico San Martino, Genova, Italy; 7 Parkinson and Movement Disorders Unit, Department of Neuroscience, University of Padua, Padua, Italy; 8 Azienda Sanitaria Provinciale Agrigento—UO Complessa Radiologia Distretto Ag 2 Sciacca-Ribera. Presidio Ospedaliero di Sciacca (AG), Sciacca, Agrigento, Italy; 9 Department of Otolaryngology Head Neck Surgery, University of Pavia, IRCCS Policlinico San Matteo Foundation, Pavia, Italy; 10 Department of Neurology, Mayo Clinic, Rochester, MN USA; 11 Clinical Nutrition and Dietetics Unit, Fondazione IRCCS Policlinico San Matteo, Pavia, Italy; 12 Gastrointestinal Physiology Laboratory, Hospital de Mataró, Universitat Autónoma de Barcelona, Mataró, Spain; 13 Centro de Investigación Biomédica en Red de Enfermedades Hepáticas y Digestivas (Ciberehd), Instituto de Salud Carlos III, Barcelona, Spain; 14 IRCCS Istituto di Scienze Neurologiche, Bologna, Italy; 15 DIBINEM, University of Bologna, Bologna, Italy; 16 Fondazione IRCCS Istituto Neurologico Carlo Besta, Milan, Italy; 17 Division of Diabetes, Endocrinology and Gastroenterology, University of Manchester, Salford Royal Hospital, Salford, UK; 18 Department of Communication Disorders, Rose Centre for Stroke Recovery and Research, University of Canterbury, Christchurch, New Zealand; 19 Department of Neuroscience “Rita Levi Montalcini”, University of Torino, Turin, Italy; 20 ENT Department, University of Padova, Padua, Italy; 21 Rehabilitation Unit, Department of Neuroscience, University of Padova, Padua, Italy; 22 Department of Speech and Language Therapy, School of Health Rehabilitation Sciences, University of Patras, Patras, Greece; 23 Parkinson’s Disease and Movement Disorders Unit, IRCCS Mondino Foundation, Pavia, Italy; 24 Department of Neurology, Oregon Health and Science University, Portland, OR USA; 25 Department of Neurology, Garibaldi Hospital, Catania, Italy; 26 Department of Public Health, Experimental and Forensic Medicine, Unit of Human and Clinical Nutrition, University of Pavia, IRCCS Mondino Foundation, Pavia, Italy; 27 Department of Sense Organs, Sapienza University of Rome, Rome, Italy; 28 Department of Clinical and Movement Neurosciences, UCL, Queen Square Institute of Neurology, London, UK; 29 University and Institute for Research and Medical Care IRCCS San Raffaele, Rome, Italy; 30 Parkinson Disease and Movement Disorders Unit, Neurology Service, Hospital Clínic de Barcelona, Institut d’Investigacions Biomèdiques August Pi i Sunyer (IDIBAPS), Centro de Investigación Biomédica en Red Sobre Enfermedades Neurodegenerativas (CIBERNED:CB06/05/0018-ISCIII), University of Barcelona, Barcelona, Spain; 31 Department of Medicine, Division of Geriatrics, Healthy Aging Center, University of Verona, Verona, Italy; 32 Department GF Ingrassia, University of Catania, Catania, Italy

**Keywords:** Parkinson's disease, Dysphagia, Swallowing disorders, Deglutition disorders

## Abstract

**Background:**

Parkinson’s disease (PD) is a neurodegenerative disorder characterized by a combination of motor and non-motor dysfunction. Dysphagia is a common symptom in PD, though it is still too frequently underdiagnosed. Consensus is lacking on screening, diagnosis, and prognosis of dysphagia in PD.

**Objective:**

To systematically review the literature and to define consensus statements on the screening and the diagnosis of dysphagia in PD, as well as on the impact of dysphagia on the prognosis and quality of life (QoL) of PD patients.

**Methods:**

A multinational group of experts in the field of neurogenic dysphagia and/or PD conducted a systematic revision of the literature published since January 1990 to February 2021 and reported the results according to PRISMA guidelines. The output of the research was then analyzed and discussed in a consensus conference convened in Pavia, Italy, where the consensus statements were drafted. The final version of statements was subsequently achieved by e-mail consensus.

**Results:**

Eighty-five papers were used to inform the Panel’s statements even though most of them were of Class IV quality. The statements tackled four main areas: (1) screening of dysphagia: timing and tools; (2) diagnosis of dysphagia: clinical and instrumental detection, severity assessment; (3) dysphagia and QoL: impact and assessment; (4) prognostic value of dysphagia; impact on the outcome and role of associated conditions.

**Conclusions:**

The statements elaborated by the Consensus Panel provide a framework to guide the neurologist in the timely detection and accurate diagnosis of dysphagia in PD.

**Supplementary Information:**

The online version contains supplementary material available at 10.1007/s00415-021-10739-8.

## Introduction

Parkinson’s disease (PD) is the second most common neurodegenerative disorder worldwide [1]. The prevalence of PD is estimated at 6.1 million individuals globally and will likely increase worldwide with increased life expectancy [2]. PD is characterized by motor features such as tremor, rigidity and bradykinesia, and several non-motor features such as dysphagia, autonomic dysfunction, sleep disorders, cognitive impairment, depression, and psychosis that may occur at any time during the disease course, but become more frequent with advanced disease [3, 4].

Dysphagia in PD is a manifestation of swallowing dysfunction that may involve oral, pharyngeal or esophageal phases of swallowing and may be present in every stage of the disease [5]. Indeed, even though swallowing disorders become apparent mostly in the advanced stage of PD, they may already be present in the early stages, when they often go undetected [5]. Dysphagia frequently worsens with disease progression and may vary with motor fluctuations [6–8]. Globally, the prevalence of dysphagia in PD is estimated between 40 and 80% depending on type of assessment performed [7].

Dysphagia can negatively affect the quality of life (QoL) of individuals because of the progressive difficulty of oral intake (food, drinks or oral medication), weight loss, dehydration, malnutrition and limitation of social activities [9]. Moreover, aspiration pneumonia due to swallowing dysfunction is an important and common cause of hospitalization in patients with PD [10, 11], resulting in severe complications and even death [12–14].

To date, there is no generally accepted approach to the screening and the diagnosis of dysphagia in PD. Therefore, there is an urgent need to identify standardized protocols for the clinical assessment and investigation of dysphagia in this population. Moreover, it is important to emphasize the need for the early identification of swallowing abnormalities in patients with PD, especially as initially they may be asymptomatic [6].

An established and formal methodology to provide reliable guidance in complex health issues in the absence of high-quality evidence is represented by a consensus-based process. In this process, expert professionals reach a consensus on statements aimed at providing a guide for clinical practice on the basis of the best available evidence and the group’s expertise. Typically, a panel of experts, after examining the relevant scientific information and discussing the clinical issues, produce statements that reflect their shared views, in agreement with available evidence [15].

To increase the awareness of dysphagia in PD in the neurological health care practice and to optimize its screening and diagnosis, a group of experts in the field of dysphagia and/or PD set forth a Multinational Consensus Conference (MCC) with the following objectives:

1. To define the appropriate timing for screening dysphagia in patients with PD and to identify reliable modalities;
2. To define appropriate investigations for detecting swallowing alterations in PD and to assess their severity;
3. To assess the impact of dysphagia on the QoL of patients;
4. To assess the prognostic values of dysphagia in PD outcome;
5. To identify unmet needs and highlight areas for future research.

## Methods

The project was initiated by the Organizing Committee during the 2018 edition of the ‘Dysphagia Update’ Meeting, an international scientific event focused on neurogenic dysphagia that has been held biannually since 2008 under the patronage of the Italian Society of Neurology, the Italian Society of Neurorehabilitation, and the European Society for Swallowing Disorders. The MCC method was designed according to the US National Institutes of Health Consensus Development Program (http://consensus.nih.gov) [16] and the Methodological Handbook of the Italian National Guideline System [17].

The project was developed over a period of 36 months following five steps: (1) assignment phase, (2) scoping phase, (3) assessment phase, (4) face-to-face MCC, held on 27–28th September 2019, at the IRCCS Mondino Foundation, and finally (5) update of the evidence (up to February 2021) and refinement of statements by e-mail.

The core of the consensus panel was formed by Italian neurologists who met regularly at the ‘Dysphagia Update’ meetings. Additional specialists, also from different medical disciplines and from other Countries were invited to achieve a broad geographic and multidisciplinary representation. Participants were selected based on their recognized involvement in the care of large cohorts of PD patients and/or their involvement in research projects on PD and/or neurogenic dysphagia, and/or because of their publication record on neurogenic dysphagia in peer-reviewed journals. Participants were invited by e-mail. A single reminder was sent to those who did not reply to the first invitation. The final group was formed by 21 neurologists, 4 ENT specialists, three phoniatricians, two gastroenterologists, 4 speech-language pathologists, 2 clinical nutritionists, one radiologist and a statistician.

In the assignment phase, four working teams with specific roles were identified:

1. The Scientific Committee, comprising seven members, planned and organized the whole project and developed the questions following the Classification of Evidence Schemes of the Clinical Practice Guideline Process Manual of the American Academy of Neurology [18]. Several clinically relevant questions were proposed by the Scientific Committee and discussed during several iterations according to the PICO format as stated in Appendix 1. The selection of the final questions to be answered in this review was also guided by the results of a preliminary literature search conducted to evaluate whether there was available evidence to support the answers. The selection of the clinical questions was ultimately driven by the following criteria: i) to identify clinically relevant, focused topics (not taking too narrow a focus nor too broad), ii) to select questions that could be answered, at least partly, on the basis of published, peer-reviewed evidence.
2. The Technical Committee, formed by six members, systematically reviewed the evidence, organized the results into tables, and assisted the other teams in all steps of the project;
3. A working group (WG) formed by nine members whose tasks were: 1) to prepare the first draft of answers to the proposed questions prior to the consensus conference and 2) to point out the research gaps in current knowledge and to propose areas for future research;
4. The consensus development panel, comprising six members, was responsible for defining the presentation procedures at the MCC and for the assessment of the final statements.

In the scoping phase, the details of the literature review necessary to answer the questions developed by the Scientific Committee (Appendix 1) were defined, together with protocol for the conference. In the assessment phase, the Technical Committee carried out a systematic review of the literature, which was reported according to PRISMA guidelines [19]. Studies eligible for inclusion were those reporting original data on patients with PD suffering from dysphagia on screening, diagnosis, prognosis and QoL, regardless of the design type, published since January 1990. The following types of studies were excluded: studies published in abstract form only, case reports, reviews, editorials, letters, studies on animals, and studies including patients with dysphagia of mixed etiology where data regarding PD could not be clearly enucleated. Published studies were identified from the National Library of Medicine's MEDLINE database, by means of specific search strategies, using a combination of exploded MeSH terms and free text (search strategy is reported in Appendix 2). Reference lists of identified articles were reviewed to find additional references. All abstracts or full papers without electronic abstracts were reviewed independently by two reviewers to identify potentially relevant studies. Disagreement was resolved by discussion. Each study was classified according to various descriptors, including topic domain, sample size, design, and level of evidence according to the Classification of Evidence Schemes of the Clinical Practice Guideline Process Manual of the American Academy of Neurology [18].

Each study was graded according to its risk of bias from Class I (highest quality) to Class IV (lowest quality). Risk of bias was judged by assessing specific quality elements (i.e., study design, patient spectrum, data collection, masking) for each clinical topic (screening, diagnosis, prognosis, treatment). This classification was performed by two reviewers, with disagreement resolved by discussion.

In consideration of the multidisciplinary of the expert groups and the generally weak strength of evidence emerged from the systematic analysis of the literature, we adopted the modified Delphi method [18] to achieve consensus and develop the final statements. The method consisted in four subsequent rounds. The first one was performed electronically: a first set of statements were generated and sent by e-mail to the experts of the WG. Answers were collected and analyzed to inform necessary changes. The second and the third rounds were carried out face-to-face, during the first and the second day of the MCC, respectively, with the participation of the entire panel. The fourth round was performed electronically: the final version of the statements, adapted, when required, according to the additional analysis of paper published since the consensus conference, was circulated by e-mail to the experts. On every round, a minimum of 80% agreement for each statement was required for inclusion in the final consensus statement (25/31).

Ultimately, the systematic literature analysis covered the period from January 1990 to February 2021.

## Results

### Questions of the consensus conference

The Scientific Committee formulated and submitted three questions each on the screening and the diagnosis of dysphagia in PD and two questions each on the QoL and the prognosis of patients with PD and dysphagia.

Questions on screening:

- When is it indicated to screen for dysphagia in patients with PD?
- When should dysphagia be suspected in patients with PD?
- What clinical tools should be used to screen for dysphagia in patients with PD?

Questions on diagnosis:

- What clinical tools should be used to detect the presence of dysphagia?
- What instrumental investigations should be used to detect the presence of dysphagia?
- How should severity of dysphagia be assessed?

Questions on QoL:

- What is the effect of dysphagia on the QoL of patients with PD?
- How should dysphagia-related QoL in patients with PD be clinically assessed?

Questions on prognosis:

- Does dysphagia influence the prognosis of PD?
- What factors or associated conditions can influence the prognosis of dysphagia in PD?

For each question, specific eliciting questions were formulated by the Technical Committee to stimulate and guide the discussion among the members of the WG (Supplementary material 1).

### Systematic review

**Fig. 1:**
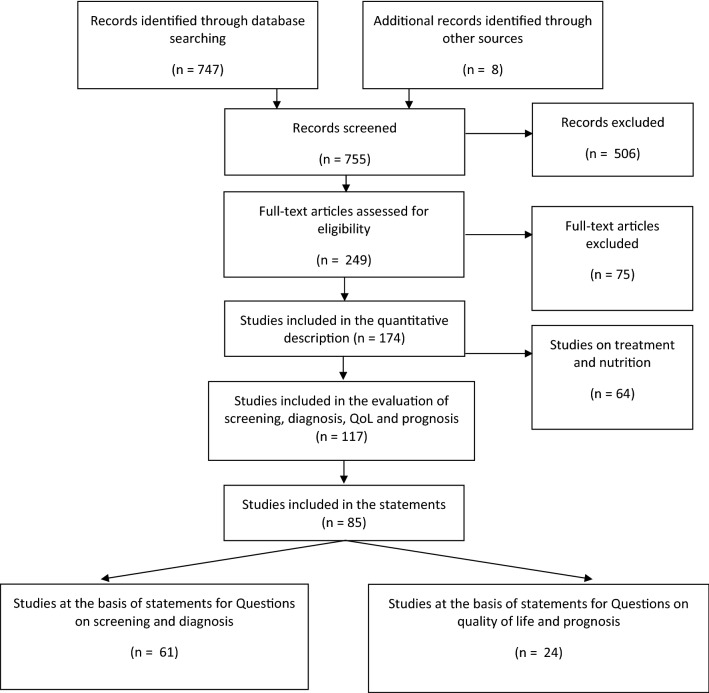
PRISMA flow diagram

The literature search retrieved 747 citations from electronic search and 8 records from manual search in the reference lists (Fig. 1). The abstract of the 747 citations were reviewed to assess potential relevance, and 249 were included for full text evaluation. A total of 174 papers finally met the prespecified inclusion criteria. Of these, 117 papers dealt with the questions posed, but only 85 contained useful findings to elaborate the statements. The majority of studies were of Class IV quality. Table 1 summarizes the main characteristics of the studies retrieved (see Supplementary material 2 for complete list of references), whereas Table 2 depicts the main information on the single studies used as the basis for the statements.

**Table 1:** Descriptive features of the 117 eligible studies included in the evaluation of screening, diagnosis, QoL and prognosis

Topic domain	All studies	Number of patients = number of studies	Cross-sectional/prospective studies	Case–control/retrospective study	Class of evidence
	*N*	*N*	*N*	*N*	N
Screening and diagnosis of dysphagia in PD	82	< 10 pts = 1 10–19 pts = 16 studies 20–50 pts = 28 studies > 50 pts = 37 studies	58	24	4 Class I 4 Class II 18 Class III 56 Class IV
QoL and prognosis of dysphagic patients with PD	35	< 50 pts = 13 50–199 pts = 13 200–500 pts = 5 > 500 pts = 4	20	15	4 Class I 8 Class II 8 Class III 15 Class IV

Studies were classified according to various descriptors, including topic domain, sample size, design, presence of diagnostic criteria of the syndrome and level of evidence according to the Classification of Evidence Schemes of the Clinical Practice Guideline Process Manual of the American Academy of Neurology ^17^. Each study was graded according to its risk of bias from Class I to Class IV (with I corresponding to the highest quality and IV to the lowest quality)

**Table 2:** Studies used as basis for the development of statements regarding questions on A) screening and diagnosis of dysphagia and B) dysphagia-related QoL and prognostic value of dysphagia in PD

A) Question	First author, y	Study design	Screening /Diagnostic test assessed	N. patients	Level of evidence
2.1 a 2.2. b	Ali, 1996	Case–control	VFSS and manometry	12	III
2.1 a	Bird, 1994	Cross-sectional	Clinical examination	16	IV
2.1 a	Hartelius, 1994	Cross-sectional	Questionnaire	258	II
2.1 a	Monteiro, 2014	Case–control	Spirometry and VFSS	30	IV
2.1 b	Buhmann, 2019	Case–control	FEES	118	IV
2.1 b	Claus, 2020	Case–control	FEES	200	III
2.1 b	Lam, 2007	Cross-sectional	Questionnaire, WST, and VFSS	45	I
2.1 b	Loureio, 2013	Case–control	Questionnaire	174	IV
2.1 b	Pflug, 2018	Case–control	FEES	122	I
2.1 b	Potulska, 2003	Case–control	Electromyography and esophageal scintigraphy	18	IV
2.1 b 2.2 b	Rodrigues, 2011	Cross-sectional	FEES	28	IV
2.1 b	Sampaio, 2014	Cross-sectional	FEES and voice recording	19	III
2.1 b	Troche, 2016	Cross-sectional	Voluntary and reflex cough and cough airflow (PEFR)	64	IV
2.1 c	Belo, 2014	Case–control	WST	10	IV
2.1 c	Buhmann, 2019	Case–control	Questionnaire	119	IV
2.1 c	Kalf, 2011	Cross-sectional	Questionnaire	178	IV
2.1 c	Manor, 2007	Cross-sectional	Questionnaire	57	III
2.1 c	Minagi, 2018	Case–control	WST, tongue pressure measurement	30	IV
2.1 c	Simons, 2014	Cross-sectional	Questionnaire	82	IV
2.1 c	Singer, 1992	Case–control	Questionnaire	48	IV
2.1 c	Vogel, 2017	Cross-sectional	Questionnaire	60	III
2.1 c	Volontè, 2002	Cross-sectional	Questionnaire	65	IV
2.2 a	Hegland, 2014	Case–control	Reflex cough testing	22	IV
2.2 a	Kanna, 2014	Case–control	WTS	100	IV
2.2 a	Mari, 1997	Cross-sectional	Questionnaire	27	II
2.2 a	Miller, 2009	Cross-sectional	WST	137	III
2.2 a	Miyazaki, 2002	Cross-sectional	WST	24	II
2.2 a	Monte, 2005	Cross-sectional	VFSS	27	IV
2.2 a	Pitts, 2010	Cross-sectional	PEFR and VFSS	58	III
2.2 a	Pitts, 2018	Cross-sectional	Tongue pressure measurement	28	IV
2.2 a	Silverman, 2016	Cross-sectional	PEFR	68	IV
2.2 a	Troche, 2014	Cross-sectional	Reflex cough testing and VFSS	20	IV
2.2 b	Alfonsi, 2007	Case–control	EKSS	28	IV
2.2 b	Argolo, 2015a	Cross-sectional	VFSS	69	IV
2.2 b	Argolo, 2015b	Cross-sectional	VFSS	71	IV
2.2 b	Bassotti, 1998	Case–control	Manometry	18	IV
2.2 b	Castell, 2001	Cross-sectional	Manometry	16	IV
2.2 b	Cosentino, 2020	Cross-sectional	Electrophysiological assessment of swallowing	19	IV
2.2 b	Ding, 2018	Cross-sectional	VFSS	116	III
2.2 b	Ellerston, 2016	Case–control	VFSS	34	IV
2.2 b	Ertekin, 2002	Case–control	Surface electromyography	58	III
2.2 b	Fuh, 1997	Cross-sectional	VFSS	19	IV
2.2 b	Gaeckle, 2019	Cross-sectional	VFSS	89	IV
2.2 b	Hammer, 2013	Cross-sectional	FEES	18	IV
2.2 b	Johnston, 1997	Case–control	VFSS and manometry	7	IV
2.2 b	Jones, 2016	Cross-sectional	VFSS	26	IV
2.2 b	Jones, 2018	Case–control	HRM	31	III
2.2 b	Kim, 2020	Cross-sectional	surface electromyography	14	IV
2.2 b	Lee, 2015	Case–control	VFSS	29	IV
2.2 b	Lee, 2019	Case–control	VFSS	23	IV
2.2 b	Moreau, 2015	Cross-sectional	VFSS	70	I
2.2 b	Nagaya, 1998	Case–control	VFSS	16	IV
2.2 b	Schiffer, 2019	Case–control	VFSS	68	IV
2.2 b	Stroudley, 1991	Cross-sectional	VFSS	24	III
2.2 b	Su, 2017	Cross-sectional	HRM	33	IV
2.2 b	Suttrup, 2017	Cross-sectional	HRM and FEES	65	IV
2.2 b	Taira, 2020	Cross-sectional	HRM	51	IV
2.2 b	Tomita, 2018	Case–control	VFSS	184	II
2.2 b	Wakasugi, 2017	Cross-sectional	VFSS	201	IV
2.2 b	Wang, 2017	Cross-sectional	EKSS	42	IV
2.2 b	Ws Coriolano, 2012	Cross-sectional	Surface electromyography	15	IV

B) Question	First author, y	Study design	Outcome measure	N. patients	Level of evidence
2.3 a 2.4 a	Akbar, 2015	Retrospective	Incidence of aspiration pneumonia; survival	5,665,710	I
2.3 a	Carneiro, 2014	Prospective	Impact of dysphagia on QoL	62	IV
2.3 a 2.4 a	Cilia, 2015	Retrospective	Survival, confinement to wheelchair or bed, fracture, PEG placement	401	II
2.3 a	Han, 2011	Prospective	Depression related to dysphagia	127	IV
2.3 a 2.3 b	Leow, 2010	Prospective	Impact of dysphagia on QoL	32	III
2.3 a 2.4 a	Lorefält, 2006	Prospective	Impact of dysphagia on QoL/Severity of dysphagia	26	IV
2.3 a 2.3 b	Manor, 2009	Prospective	Mood changes related to dysphagia	69	II
2.3 a	Miller, 2006	Retrospective	Impact of dysphagia on QoL	137	III
2.3 a	Plowman-Prine, 2009	Prospective	Impact of dysphagia on QoL	36	III
2.3 a	Silbergleit, 2012	Prospective	Impact of dysphagia on emotional changes	14	IV
2.3 a 2.3 b	Storch, 2013	Prospective	Impact of dysphagia on QoL	100	IV
2.3 a 2.3 b	Van Hooren, 2016	Prospective	Impact of dysphagia on emotional changes	100	IV
2.4 a	Auyeung, 2012	Retrospective	Survival	171	II
2.4 a	Fabbri, 2019	Retrospective and Prospective Cross-sectional	Survival, institutionalization	50	I
2.4 a	Hussain, 2018	Retrospective	Survival	51	III
2.4 a	Lo, 2009	Retrospective	Survival	573	II
2.4 a	Malmgren, 2011	Retrospective	Survival	191	III
2.4 a	Müller, 2001	Retrospective	Survival	17	II
2.4 a	Robbins, 2008	Prospective	Pneumonia	255	I
2.4 a	Barichella, 2013	Prospective	Nutritional status	208	I
2.4 a	Cereda, 2014	Retrospective	Non-motor symptoms	6462	IV
2.4 a	Goh, 2016	Retrospective	Pneumonia and choking	194	II
2.4 a	Lee, 2016	Prospective	Pneumonia	66	IV
2.4 a	Miller, 2009	Prospective	Impact of dysphagia on PD	137	III

Studies were classified according to various descriptors (e.g., study design, presence or not of a reference standard diagnostic test, sampling method, sample size, blinding, presence of clearly stated inclusion and exclusion criteria) according to the Classification of Evidence Schemes of the Clinical Practice Guideline Process Manual of the American Academy of Neurology ^17^. Each study was graded according to its risk of bias from Class I to Class IV (with I corresponding to the highest quality and IV to the lowest quality)

*EKSS* Electro-Kinesiologic Swallowing Study; *FEES* Fiberoptic endoscopic evaluation of swallowing; *HRM* High-resolution manometry; *PEG* Percutaneous Endoscopic Gastrostomy; *PEFR* Peak expiratory airflow rate; *VFSS* Videofluoroscopic study of swallowing; *WST* Water swallow test

### Screening of dysphagia in PD

Although dysphagia is a common symptom in PD, most patients with PD do not complain of swallowing difficulties even when specifically asked, because they are generally not aware of their swallowing problems [20, 21].

Therefore, there is a gap in the literature on dysphagia prevalence between subjectively reported impairment (35%) and objectively confirmed swallowing impairments by screening questionnaires or clinical evaluations (85%) [7, 22]. Indeed, signs of penetration and/or aspiration may be detected in 20% of the PD population without any complaint of swallowing difficulties [23, 24].

The early diagnosis of dysphagia in PD may be challenging. However, many symptoms and signs may indicate the need for a screening. These are: increased duration of meals, difficulty in tablet swallowing and sensation of food sticking or persisting in the throat after swallowing, coughing and choking during ingestion of food and liquids, post-swallowing changes in voice (e.g. gurgling voice), weight loss or low body mass index while recurrent chest infections [25–29]. Also, drooling is considered by some authors as a possible indirect marker of dysphagia, since it can be the consequence of oropharyngeal swallowing alterations [30]. Using a modified barium swallowing videofluoroscopy, Nóbrega et al. [30] showed that all patients with PD and drooling also presented changes in the oral phase of swallowing, whereas alterations in the pharyngeal phase were detected in 94% of subjects. Drooling also correlates with dysphagia severity and may have a negative prognostic value, as it is associated with an increased risk of salivary aspiration without any outward sign of coughing, choking, or respiratory change (silent aspiration) [30–32].

A recent study [33] conducted in patients with PD aged > 63.5 years identified the following characteristics as predictors of an increased risk to develop dysphagia: a daily levodopa equivalent dose higher than 475 mg and a PD clinical subtype characterized by early postural instability and gait difficulty.

Altogether, the data from the literature suggest that dysphagia in PD should be suspected in the presence of direct symptoms (coughing or choking when eating or drinking, wet-sounding voice when eating or drinking, sensation of food stuck in the throat and difficulty of chewing food properly), or indirect signs (congestion of the lower respiratory tract, bronchitis or pneumonia and unintentional weight loss).

In these cases, a screening evaluation should be completed regardless of PD disease stage [5, 34].

The presence of different symptoms and/or signs in combination can increase the sensitivity and specificity of the screening procedures [35, 36].

Different clinical scales and questionnaires have been proposed for screening dysphagia in neurodegenerative diseases [35–45], though there is no agreement on the best algorithm for evaluating patients with PD.

The following clinical scale and questionnaires (evidence of class III and IV) may be used for screening of dysphagia in PD:

- Swallowing disturbance questionnaire (SDQ) [38]: a self-reported questionnaire containing 15 items on swallowing disturbances. It is considered a validated tool to detect early dysphagia in patients with PD. It has a good sensitivity and specificity (80.5 and 81.3%, respectively).
- Munich Dysphagia test-Parkinson’s disease (MDT-PD) [39]: a self-reported questionnaire useful to screen for initial oropharyngeal symptoms and the risk of laryngeal penetration and/or aspiration in PD. It consists of 26 items divided into 4 categories. The MDT-PD is considered a sensitive and specific questionnaire; however, it was reported that compared with FEES it has a low sensitivity in detecting aspiration, though maintaining good specificity [46].
- Swallowing Clinical Assessment Score in Parkinson’s disease (SCAS-PD): a quantitative clinical scale consisting of 12 items designed to detect alterations in the oral and pharyngeal phases of swallowing in PD [36]. Branco et al. [40] validated this scale using the videofluoroscopic swallow study (VFSS) as the reference diagnostic test. They showed that SCAS-PD has a high sensitivity (100%) and specificity (87.5%) and it enables to detect clinical signs of aspiration with a good concordance with the gold standard VFSS (weighted kappa concordance rate of 0.71).
- Radboud Oral Motor Inventory for Parkinson’s disease (ROMP) [41]: a questionnaire developed to assess three main domains: speech, swallowing, and saliva control. This scale represents a valid tool to identify swallowing difficulties in PD, though it contains a limited number of items.
- Handheld Cough Testing (HCT), a novel tool for cough assessment and dysphagia screening in PD. The HCT is able to identify differences in cough airflow during reflex and voluntary cough tasks, and screen for people with dysphagia in PD with high sensitivity and specificity (90.9% sensitivity; 80.0% specificity)[47].

Although these screening tools look promising for an early diagnosis of dysphagia in patients with PD, they are not cross-culturally validated. Therefore, there is an unmet need to translate, adapt, and cross-culturally validate screening measures.

Box 1. Recommendations on the screening of dysphagia in PD

**Table:** 

**a. When is it indicated to screen for dysphagia in patients with PD?** The statement is based on core literature consisting of Class II [21], III [23] and IV [20, 24] level studies and expert opinion. -The search for symptoms or signs that are suspicious for the presence of dysphagia is recommended at the first neurologic visit. If symptoms or signs are detected, a screening test is always recommended. Re-evaluations are recommended at every follow-up visit, preferably at least once a year. **b.** **When should dysphagia be suspected in patients with PD?** Statements are based on core literature consisting of Class I [5, 35], III [28, 33] and IV [27, 29–31, 34, 36] level studies and expert opinion. - In the presence of at least one of the conditions listed below: Increased eating time (meal duration), post-swallowing coughing, post-swallowing gurgling voice, drooling, choking, breathing disturbance, unintentional weight loss, difficulty to swallow pills, sensation of retention of food, pneumonia episode(s). - In patients who answer ‘yes’ to either of the following questions: "have you experienced any difficulty in swallowing food or drink?" "have you ever felt choked with food?" *Comment:* The risk of dysphagia increases with the number of symptoms or signs observed, age, and disease progression.
**c.** **What clinical tools should be used to screen for dysphagia in patients with PD?** Statements are based on core literature consisting of Class III [38, 43] and IV [36, 37, 39, 41, 42, 44–46] level studies and expert opinion. - The swallowing disturbance questionnaire (SDQ) represents the most appropriate self-reported patient test for screening swallowing disorders in PD. -The MDT-PD test, SCAS-PD and ROMP may be also considered valid questionnaire-based tools for dysphagia screening in PD. - Positive results at a screening test impose further investigation with diagnostic tests to confirm the presence of dysphagia and to assess its severity. *Comment:* A thorough medical history evaluation is also needed to complete the screening assessment of dysphagia in PD.

### Diagnosis of dysphagia in PD

The clinical swallowing examination has a higher sensitivity to identify swallowing abnormalities compared to screening questionnaires [6, 24]. Thus, it is indicated in all patients with a positive screening test result. The clinical swallowing examination is performed preferably by a speech-language pathologist and includes a patient/caregiver interview, evaluation of cognition and communication abilities, oral motor assessment, and swallowing trials. For those centres who do not have a speech-language therapist with an expertise in the evaluation of neurogenic dysphagia, a referral pathway should be put in place.

The Water Swallow Test (WST) is among the most common tools used during clinical swallowing examination because of its rapidity and ease of use. The WST may detect dysphagia early in PD [48] and identify subjects at risk of aspiration [49]. However, it may underestimate the incidence of oropharyngeal dysphagia, since its diagnostic accuracy depends on the preservation of the cough reflex and pharyngo-laryngeal sensitivity. Therefore, the combination of the WST with clinical tests assessing voluntary and/or reflex cough function increases the positive and negative predictive value of the clinical swallowing assessment [50, 51].

In addition to the above, cough testing can be useful for assessing severity of dysphagia and aspiration risk [27, 52]. In particular, compared to voluntary cough testing, reflexive cough testing can be more sensitive in distinguishing between patients with PD with mild (PAS 3–5) or severe dysphagia (PAS 6–8), as well as between patients with penetration above (PAS 2–3) or below (PAS 4–8) the level of the vocal folds [27].

Two studies have shown that reduced tongue pressure and motility are among other possible indicators of early dysphagia in PD [42, 53].

Several instrumental investigations detect swallowing abnormalities with higher sensitivity than the clinical swallowing examination. Swallowing abnormalities of swallow have been observed in almost all patients using different diagnostic techniques, even in the early stages of disease in studies performed by different groups [23, 24, 54–59]. There is no consensus on whether these investigations should be carried out in all patients with PD regardless of the presence of signs and symptoms associated with dysphagia. This represented one of the main subjects of debate during the MCC. On one hand, when considering that silent aspiration may go undetected by clinical evaluation and it may be present even in the early stages of the disease, it seems reasonable to apply instrumental investigations also in subjects with clinically safe and functional swallowing, [5, 20, 58]. On the other hand, it seems important to consider that some instrumental diagnostic investigations for dysphagia are not widely available. Thus, the prevailing view was to consider mandatory such evaluation only in patients with clinical signs of dysphagia (see statement below).

Several studies have evaluated the role of different investigation methods in the diagnosis of dysphagia in PD. VFSS and FEES should be used as a first approach, as they enable detection of aspiration, penetration, and residue with similar high sensitivity and specificity [60]. VFSS directly reveals penetration/aspiration [61–63], also providing useful information when the oral and/or esophageal phase is impaired [64–68]. FEES ensures an appropriate assessment of the pharyngeal phase of swallowing and the detection of penetration/aspiration phenomena, both directly (before or after the swallow, i.e. in case of premature spillage and residue, respectively) and indirectly [5, 31, 69–71]. FEES has the advantage over VFSS of being easier to perform, even at the patient’s bedside, and allows to test swallowing of real food and to assess secretion management. Moreover, as FEES does not require the use of radiation, it can be repeated even at short time intervals, thus allowing accurate follow-ups.

Oro-Pharyngo-Esophageal Scintigraphy (OPES) is a useful tool for the early detection of dysphagia [72], but its diagnostic role in PD remains to be ascertained as only a single study has been carried out in this patient population [73]. Although only preliminary evidence is available, swallowing evaluation with high-resolution manometry (HRM) represents another interesting diagnostic tool, capable to show subtle swallowing changes in the early stages of PD even in the absence of swallowing changes on VFSS [57, 74].

The Electro-Kinesiologic Swallowing Study (EKSS) represents an additional useful diagnostic tool to explore the pathophysiological mechanisms of oropharyngeal dysphagia and provide clues for treatment selection [56, 75–77]. For example, electromyography of the cricopharyngeal muscle (i.e., the main component of the upper esophageal sphincter, UES) helps to clarify whether failure in UES opening during the pharyngeal phase of swallowing is due to persistent sphincter hyperactivity or to reduced elevation of the pharyngeal-laryngeal structures [78, 79]. In the former case, botulinum toxin injection into the cricopharyngeal muscle may be beneficial [78, 79]. However, very few centers use UES electromyography routinely, thus this examination cannot currently be considered as a standard test for dysphagia.

Gastroenterological investigations including esophageal manometry, upper gastrointestinal endoscopy, acid- and reflux-related tests, and/or radiological investigations such as barium swallow should always be carried out in the presence of esophageal symptoms (e.g. dysphagia for solid foods, regurgitation, food sticking after swallowing) and/or when oropharyngeal evaluations detect findings suggestive of structural or functional deficits in the esophagus, including a Zenker’s diverticulum, a neoplasm or an esophageal motility dysfunction [58, 80]. In recent years, pharyngo-esophageal HRM has provided useful insights into the process of swallowing by enabling the detection of esophageal dysmotility, with particular interest in upper and lower esophageal sphincters [81, 82]. Manometry studies, often in combination with VFSS or FEES, have shown that dysphagia in PD is associated with a high prevalence of esophageal motility disturbances [57, 80, 83, 84]. However, the impact of these alterations on swallowing and their role in influencing the clinical management of dysphagia remain to be clarified [58].

Clinicians should keep in mind that findings from clinical and instrumental investigations of swallowing may not be entirely representative of the patient’s eating and drinking performance in real life. Variables such as distractions, specific consistencies and bolus size, medications, motor fluctuations and dyskinesias may impact the testing results [85, 86]. Thus, it is important to observe patients in their usual eating and drinking habits, or alternatively gather this information in the case history or with questionnaires [87]. The therapeutic effect of the anti-parkinsonian drugs on swallowing function is highly variable and can affect results from swallowing investigations. Though the dopaminergic medications might improve dysphagia in some patients, in others, they either showed no effect or negatively affected the swallowing function, also depending on the stage of the disease [88–90]. Similarly, studies testing the effect of deep brain stimulation (DBS) on swallowing function have yielded conflicting findings, showing beneficial, absent or detrimental effects [91].

Once the diagnosis of dysphagia is provided, standardized methods to assess swallowing severity should be carried out to guide the best treatment strategy and for prognostic assessment. Most of the studies conducted in patients with PD have adopted the Penetration-Aspiration Scale (PAS) [92]. A few studies have used other validated scales such as the Dysphagia Outcome and Severity Scale (DOSS) [93], and the Functional Oral Intake Scale (FOIS) [94].

Box 2. Recommendations on the diagnosis of dysphagia

**Table:** 

**a.** **What clinical tools should be used to detect the presence of dysphagi**a? Statements are based on core literature consisting of Class II [49, 50], III [6, 95] and IV [24, 27, 42, 48, 50–53, 85, 96, 97] level studies and expert opinion. -PD patients with a positive screening for dysphagia should undergo an in-depth clinical swallowing examination by a speech-language pathologist with special training in swallowing disorders. If a speech-language therapist with an expertise in the evaluation of neurogenic dysphagia is not available on site, a referral pathway should be put in place. -The clinical swallowing examination should include: 1) a thorough examination of cranial nerves; 2) the evaluation of dry swallows; 3) on-command and/or reflexive cough testing; 4) the evaluation of swallowing of various food and liquid consistencies; and 5) the detection of possible signs or symptoms of reduced swallowing efficiency and safety. Assessment of cognition and speech should always be carried out in conjunction with the clinical swallowing examination. -In PD patients with motor fluctuations, the swallowing examination should be performed during an ON phase. In the presence of cervical-cranial dyskinesias, clinical evaluation should preferably be conducted during both phases (ON or OFF) to identify the safest moment for the patient to eat or drink. The clinical examination should not be performed during exacerbation periods of cervical-cranial dyskinesias interfering with the ability of feeding. -Meal observation, assessing a higher number of swallowing acts and including information on feeding dependency and meal duration, can provide valuable information on swallowing function. However, this is often not feasible in the outpatient setting. In these cases, we recommend gathering information about typical eating/drinking patterns and experiences by clinical history or questionnaires. - Patients with DBS implants should be tested in an ON medication phase with the stimulator turned ON. In case of a strong suspicious of detrimental effects of DBS on swallowing, the patient should be assessed in both conditions: with the stimulator turned ON and with the stimulator OFF. Assessment in both conditions should be performed after an adequate interval of time (generally several hours) to allow for the full array of motor and non-motor features to manifest. Different combinations of the DBS/medication states should be also tested in selected patients in which detrimental interactions between different DBS and medication states are suspected. **b**. **What instrumental investigations should be used to detect the presence of dysphagia**? Statements are based on core literature consisting of Class I [5, 98], II [62], III [23, 55, 61, 74, 75] and IV [20, 31, 56–59, 63–69, 73, 76, 77, 80, 82–84, 99–105] level studies and expert opinion. - When the clinical evaluation suggests the presence of dysphagia, patients should undergo an instrumental investigation for the assessment of swallowing. Depending on local availability and on specific advantages of each method, either FEES or VFSS are recommended as first-line diagnostic tools. - On suspicion of esophageal disorders, patients should be referred for further investigations such as upper gastrointestinal endoscopy, barium swallow, esophageal manometry, and/or acid- and reflux-related tests. - If impaired motility of the upper esophageal sphincter is suspected based on FEES or VFSS, pharyngo-esophageal manometry (possibly with the high-resolution modality) and/or electromyographic examination of the cricopharyngeal muscle should be considered. - The electrophysiological evaluation of oropharyngeal swallowing might provide further insights into the pathophysiological basis of dysphagia in PD and give useful clues for treatment.
**c**.** How should severity of dysphagia be assessed?** In the literature, there are no validated scales specific for PD to rate dysphagia severity. The following statement is, therefore, entirely based on expert opinion. - Several scales exist to rate the severity of neurogenic dysphagia. The most widely used and available in multiple languages are PAS, FOIS and DOSS. PAS is based on imaging data, FOIS on clinical assessment, DOSS on both clinical and instrumental parameters.

### Relevance of dysphagia for the quality of life of patients with PD

Dysphagia negatively affects QoL in PD. The progression of swallowing difficulties may cause choking, coughing or breathing problems during the meal, and often leads to dietary restrictions and prolonged meal duration. Altogether, these changes have an important psychosocial burden for patients with PD because they may discourage or impair social activities and relationships [106–109]. Swallowing problems negatively influence wellbeing, self-confidence and social integrations [108], which in turn result in frustration and isolation. Depression is indeed frequently associated with reduced QoL in patients with PD with swallowing disorders [107, 110–112].

Moreover, difficulty in taking oral anti-parkinsonian therapy with consequent worsening of motor and non-motor symptoms is another problem that frequently affects patient’s QoL [113].

In the late stages of the disease, due to severe dysphagia, PEG placement becomes mandatory when medium/long-term enteral feeding is needed to prevent malnutrition, weight loss and aspiration. This interventional procedure is often not well accepted by the patients and their caregivers and may thus have a further negative impact on QoL [108, 114].

Though not specific for PD, the Swallowing Quality of Life (SWAL-QOL) is the most widely used questionnaire to assess the impact of dysphagia on the QoL of patients with PD both in the literature and in the clinical practice [107, 109, 115]. SWAL-QOL [116] assesses different aspects of the swallowing function that are experienced by the patient (i.e., food selection, social functioning, fear, eating duration, eating desire, communication) with a recall period of 1 month. The questionnaire is available and validated in several languages.

The 39-item Parkinson’s disease Questionnaire (PDQ-39) and the short-form 8-item Parkinson’s disease Questionnaire (PDQ-8) are also used in PD to assess the psychosocial impact of dysphagia on QoL, but they are not specific for swallowing disturbances [117].

A limited number of studies have investigated the relationship between dysphagia severity and the impact on QoL [106, 112, 115, 118, 119]. The severity of dysphagia significantly affects QoL and the progression of the disease [12, 13, 120], though this relationship is not linear. In the study by Leow et al. [106], QoL deterioration was proportional to the progression of dysphagia, and the subjects who required diet modifications presented with significantly reduced SWAL-QOL scores.

Box 3. Recommendations on the relevance of dysphagia for QoL in PD

**Table:** 

**a.** **What is the impact of dysphagia on QoL of patients with PD?** Statements are based on core literature consisting of Class I [12], II [13, 107], III [106, 108, 112] and IV [109, 111, 113, 115, 117, 118] level studies and expert opinion. -Dysphagia affects the QoL of patients with PD. -Dysphagia severity seems to correlate with poorer QoL. -The three main domains of QoL affected by dysphagia in PD patients are: - loss of the social aspect of eating; - loss of personal autonomy; - difficulties in taking oral therapy.
**b. How should dysphagia-related QoL in patients with PD be clinically assessed?** Statements are based on core literature consisting of Class II [107], III [106] and IV [109, 115–117] level studies and expert opinion. - In the absence of validated dysphagia-related QoL scales for PD, the SWAL-QOL scale can be used for the purpose PDQ39 is a validated scale for QoL in PD and can be used for indirectly evaluating the impact of dysphagia in PD. - Patients’ cognitive abilities should be considered when using questionnaires for assessing QoL.

#### Prognostic value of dysphagia and prognostics factors for dysphagia in PD

In this paragraph, we focus on the impact of dysphagia on comorbidities and life expectancy of subjects with PD, and on the factors that are associated to dysphagia severity.

The incidence of aspiration pneumonia is more than three-fold higher in PD subjects than age-matched controls [12], and patients with dysphagia are more likely to die of pneumonia than those with normal swallowing [121]. Presence of comorbidities (e.g., chronic respiratory and cardiovascular diseases, cerebrovascular disease, chronic renal or liver disease) and lower level of compliance to enteral feeding in PD patients with severe dysphagia increases the risk of pneumonia and choking [122]. Weight loss is more frequent in patients with PD with eating problems [113], and the risk of malnutrition appears to be dependent on dysautonomic symptoms including dysphagia [9].

Survival after onset of dysphagia is poor in PD [120]. Moreover, the severity of dysphagia represents the most important prognostic factor for the occurrence of death in the later stages of the disease. [13, 14] Pneumonia represents the most common cause of death in PD [123, 124]. Aspiration of solid food, liquids, saliva or gastric contents represents the leading cause of pneumonia in this patient population, [11] and it is significantly associated with survival from diagnosis [125].

As regards the factors that may be associated to dysphagia severity, disease progression is associated with more severe swallowing difficulties. [6, 41, 126] Furthermore, an impaired cough response has a negative impact on dysphagia severity [52]. This agrees with the notion that integrity of pharyngeal–laryngeal sensitivity and cough efficiency influence the risk of aspiration pneumonia, as reflex and voluntary cough are important mechanisms of airway protection during swallowing [27, 127]. The association between pneumonia and reduced oral hygiene is well documented [128, 129]. However, we did not find any specific evidence regarding the prognostic value of reduced oral hygiene on dysphagia and/or PD outcome. Yet, it is conceivable that poor oral hygiene may have a negative impact on dysphagic patients with PD. Finally, a relationship between cognitive impairment, dysphagia severity and risk of aspiration pneumonia has been shown in PD [6, 130].

Box 4.Recommendations on prognostic value of dysphagia in PD

**Table:** 

**a.** **Does dysphagia influence the prognosis of the PD?** Statements are based on core literature consisting of Class I [12, 14, 126], II [13, 120–122, 124], and III[125, 131] and IV [113, 130, 132] level studies and expert opinion. The presence of dysphagia negatively influences the prognosis of patients with PD. The presence of dysphagia, and more specifically, anterograde aspiration in the lungs is strongly correlated to a higher risk of choking and aspiration pneumonia. Poor oral care, load of comorbidities and cognitive impairment are possibly associated to a worse prognosis in dysphagic PD patients.
**b**.** What factors or associated conditions can influence the prognosis of dysphagia in PD?** Long duration and greater severity of PD has a negative impact on the swallowing function. An impaired cough response has a negative impact on dysphagia severity.

### Limitations of the study

Some limitations of the present work are worth mentioning. The first regards the recruitment of participants, which, in the absence of a strictly codified methodology for selection, was based on practical considerations and on their voluntary acceptance of our invitation. This approach led to the formation of an expert group with a prevalent representation of neurologists compared to other specialists, and a prevalence of Italian specialists compared to specialists from other countries. Thus, it is possible that the statements elaborated by this panel group do not reflect entirely the point of view of the wider international medical and scientific communities. It is worth noting, however, that we put in place several measures to involve as many experts in the field as possible and that, once the panel was created based on voluntary adhesion, the ruling process was supported by a thorough revision of the data available from the literature. This approach seemed the best compromise between spending more time in trying to include a larger group of experts and the need to deliver this consensus in an acceptable time frame to provide timely guidance to clinician on a critical issue in the management of PD patients. Another shortcoming of our consensus statements is that PD patients, their carers or representatives were not involved in the process. Indeed, their contribution would have certainly been relevant, especially as regards the quality of life topic .

### Unmet needs, areas for future research

This consensus process identified several critical areas for the timely and correct diagnosis of dysphagia in PD that could not be properly addressed by the panel of experts due to the lack of reliable evidence. Future studies need to focus on the reliability of clinical methods to screen and assess dysphagia in PD and should better define the conditions when instrumental investigations are required. Other areas worth attention are the development of validated all-inclusive (clinical and instrumental) scales for rating dysphagia severity in PD, and of PD-specific tools to assess the impact of dysphagia on QoL.

In particular, there was agreement among the consensus participants that future research should aim to address the following additional questions:

1. What are the pathophysiological elements of dysphagia in PD and their neurophysiological correlates in terms of oropharyngeal sensory and motor impairment?
2. What is the role played by esophageal dysmotility in PD?
3. In which way malnutrition and dehydration in PD affect dysphagia severity and the risk of complications?
4. Does PEG insertion change the prognosis and QoL of patients with severe dysphagia?
5. Are there useful biomarkers for the early detection of PD-related dysphagia, e.g. levels of substance P in saliva?[133]
6. How should we choose the best timing of clinical and instrumental evaluation in relation to the motor fluctuation of patients in specific situations (severe dyskinesias, DBS, etc.)?
7. Are the biomechanical or pathophysiological elements of dysphagia in PD affected by different anti-parkinsonian treatment strategies or different complications?

### Electronic supplementary material

Below is the link to the electronic supplementary material.

Supplementary file1 (DOCX 19 KB)

Supplementary file2 (DOCX 42 KB)

## Appendix 1. Research questions based on PICO

### Participants/population

Patients with Parkinson’s disease.

### Intervention(s), exposure(s)

Questions on screening and diagnosis: presence and absence of oropharyngeal dysphagia, and any diagnostic test or screening test.

Questions on prognosis: oropharyngeal dysphagia as exposures.

Question on treatment: any treatment of oropharyngeal dysphagia.

### Comparator(s)/control

Not applicable.

### Main outcome(s)

The primary outcome of the systematic review is to gather evidence on: (1) screening approach to oropharyngeal dysphagia in PD; (2) definition of the diagnostic criteria of oropharyngeal dysphagia in PD; (3) definition of the prognostic value of oropharyngeal dysphagia on QoL and PD outcome.

Such evidence will inform the statements of a Multinational Consensus Conference to provide guidance to clinicians on the above listed topics.

## Appendix 2. Search strategy on MEDLINE

("deglutition disorders"[MeSH] OR ("deglutition disorder"[All Fields] OR "deglutition disorders"[All Fields] OR "swallowing disorders"[All Fields] OR "swallowing disorder"[All Fields] OR ("deglutition disorders"[MeSH Terms] OR ("deglutition"[All Fields] AND "disorders"[All Fields]) OR "deglutition disorders"[All Fields] OR "dysphagia"[All Fields]))).

AND

("Parkinson Disease"[Mesh] OR ("Parkinson's disease"[All Fields] OR "Parkinson disease"[All Fields] OR Parkinson[All Fields])).
