# Supplementary material for: A multinational consensus on dysphagia in Parkinson's disease: screening, diagnosis and prognostic value
Source: J Neurol. 2021 Aug 21;269(3):1335–52. doi: 10.1007/s00415-021-10739-8 (PMC8857094; doi:10.1007/s00415-021-10739-8)
Supplement: Supplementary file 1 — Supplementary file1 (DOCX 19 KB) [file 415_2021_10739_MOESM1_ESM.docx]

**Supplementary material (1).** Questions posed to Workgroup 1 including eliciting questions provided to encourage discussion.

**SCREENING**

**2.1 a) When is it indicated to screen for dysphagia in patients with Parkinson’s Disease (PD)?**

- At what stage of the disease should patients with PD be screened for dysphagia?
- If the screening is negative, how often should be patients with PD reassessed for the presence of dysphagia?

**b) When should dysphagia be suspected in patients with PD?**

- Are there specific clinical history elements to suspect the presence of dysphagia in patients with PD? For example, concurrent symptoms such as dysarthria or drooling?

**c) What clinical tools should be used to screen for dysphagia in patients with PD?**

- What is the clinical algorithm to apply for the screening of dysphagia in patients with PD?
- Which aids/tools should be suggested for the screening of dysphagia in PD (e.g. questionnaires, water swallow test, low-tech methods including electrophysiological assessment of oropharyngeal swallowing, assessment of maximal tongue pressure, reflex cough assessment, acoustic analysis of swallowing, etc.)?

**DIAGNOSIS**

**2.2 a) What clinical tools should be used to detect the presence of dysphagia?**

- Which methods of clinical evaluation should be used in PD patients who tested positive at the screening tests to establish the presence of dysphagia and to characterize its pathophysiological mechanisms?

**b) What instrumental investigations should be used to detect the presence of dysphagia?**

- Which instrumental method of swallowing assessment should be performed first (e.g., FEES, VFSS, electrophysiological assessment)? Are there specific signs or symptoms that should guide this choice in patients with PD?
- Should the instrumental assessment of swallowing be carried out in any case (i.e. to detect subclinical abnormalities) or only when the clinical assessment is suggestive of swallowing abnormalities?
- In PD, are there specific signs or symptoms that may indicate the need to perform specific clinical and/or instrumental evaluations? For example, are there any specific indications to perform VFSS, oropharyngoesophageal scintigraphy, esophageal manometry, spirometric assessment or peak expiratory (cough) airflow rate (PEFR) evaluation, etc?
- Are there methods that can be sufficient when used alone, e.g. FEES?

**c) How should severity of dysphagia be assessed?**

- Once the diagnosis of dysphagia is made, are there specific clinical and/or instrumental methods that should be used to assess prognosis and guide the best treatment strategy?

**QUALITY OF LIFE**

**2.3 a) What is the effect of dysphagia on QoL of patients with PD?**

- Which are the most frequent aspects that could affect QoL in patients with PD and dysphagia? For example: depressive symptoms due to the perception of a loss in personal autonomy; difficulties in tolerating an adapted diet or a PEG placement, difficulties in taking dopaminergic therapy with consequent worsening of motor and non-motor symptoms, limitation of social activity.
- Is the severity of dysphagia proportional to the degree of deterioration in QoL?

**b) How should dysphagia-related QoL in patients with PD be clinically assessed?**

- Which are the most appropriate tools/scales for assessing the impact of dysphagia on the patient's QoL?

**PROGNOSIS**

**2.4 a) Does dysphagia influence the prognosis of PD?**

- What is the frequency of penetration-aspiration in dysphagic PD patients?
- What clinical and/or instrumental findings could be useful for prognostic assessment?
- What is the impact of the efficiency of dysphagia on the prognosis of PD? What is the impact of voluntary and reflex cough production on the airway safety of patients with PD and dysphagia?

**b) What factors or associated conditions can influence the prognosis of dysphagia in PD?**

- What could be the impact of any comorbidities on dysphagia and on the prognosis of patients with PD and dysphagia?
