# Supplementary material for: A multinational consensus on dysphagia in Parkinson's disease: screening, diagnosis and prognostic value
Source: J Neurol. 2021 Aug 21;269(3):1335–52. doi: 10.1007/s00415-021-10739-8 (PMC8857094; doi:10.1007/s00415-021-10739-8)
Supplement: Supplementary file 2 — Supplementary file2 (DOCX 42 KB) [file 415_2021_10739_MOESM2_ESM.docx]

**Supplementary material (2).** List of references included in the quantitative description and provided to the working group for statements development.

**SCREENING AND DIAGNOSIS**

Alfonsi E, Versino M, Merlo IM, Pacchetti C, Martignoni E, Bertino G, et al. Electrophysiologic patterns of oral-pharyngeal swallowing in parkinsonian syndromes. Neurology [Internet]. 2007 Feb 20 [cited 2019 May 22];68(8):583–9. Available from: http://www.neurology.org/cgi/doi/10.1212/01.wnl.0000254478.46278.67

Ali GN, Wallace KL, Schwartz R, DeCarle DJ, Zagami AS, Cook IJ. Mechanisms of oral-pharyngeal dysphagia in patients with Parkinson’s disease. Gastroenterology [Internet]. 1996b Feb [cited 2020 Apr 18];110(2):383–92. Available from: <http://www.ncbi.nlm.nih.gov/pubmed/8566584>

Argolo N, Sampaio M, Pinho P, Melo A, Nóbrega AC. Swallowing disorders in Parkinson’s disease: Impact of lingual pumping. Int J Lang Commun Disord [Internet]. 2015a Sep 1 [cited 2020 Apr 18];50(5):659–64. Available from: http://www.ncbi.nlm.nih.gov/pubmed/25652413

Argolo N, Sampaio M, Pinho P, Melo A, Nóbrega AC. Videofluoroscopic Predictors of Penetration-Aspiration in Parkinson’s Disease Patients. Dysphagia [Internet]. 2015b Dec 1 [cited 2020 Apr 18];30(6):751–8. Available from: <http://www.ncbi.nlm.nih.gov/pubmed/26492880>

Bartlett RS , Kenz MK, Wayment HA, Thibeault SL. Correlation Between EAT-10 and Aspiration Risk Differs by Dysphagia Etiology. Dysphagia. 2021 Jan 24. doi: 10.1007/s00455-021-10244-0. Epub ahead of print. PMID: 33486590

Bassotti G, Germani U, Pagliaricci S, Plesa A, Giulietti O, Mannarino E, et al. Esophageal manometric abnormalities in Parkinson’s disease. Dysphagia. 1998;13(1):28–31.

Belo LR, Gomes NAC, Coriolano MDGWDS, et al. The relationship between limit of dysphagia and average volume per swallow in patients with Parkinson’s disease. Dysphagia. 2014;29:419–424.

Bird M, Woodward M, Gibson E, Phyland D, Fonda D. Asymptomatic swallowing disorders in elderly patients with Parkinson’s disease: a description of findings on clinical examination and videofluoroscopy in sixteen patients. Age Aging. Epub 1994.:251–254.

Borders JC, Brandimore AE, Troche MS. Variability of Voluntary Cough Airflow in Healthy Adults and Parkinson's Disease. Dysphagia. 2020 Sep 25.

Buhmann C, Bihler M, Emich K, et al. Pill swallowing in Parkinson’s disease: A prospective study based on flexible endoscopic evaluation of swallowing. Park Relat Disord. 2019;62:51–56.

Castell JA, Johnston BT, Colcher A, Li Q, Gideon RM, Castell DO. Manometric abnormalities of the oesophagus in patients with Parkinson’s disease. Neurogastroenterol Motil [Internet]. 2001 Aug [cited 2020 Apr 18];13(4):361–4. Available from: <http://www.ncbi.nlm.nih.gov/pubmed/11576395>

Clarke CE, Gullaksen E, Macdonald S, Lowe F. Referral criteria for speech and language therapy assessment of dysphagia caused by idiopathic Parkinson's disease. Acta Neurol Scand. 1998;97(1):27‐35. doi:10.1111/j.1600-0404.1998.tb00605.x

Claus I, Muhle P, Suttrup J, Labeit B, Suntrup-Krueger S, Dziewas R, Warnecke T. Predictors of Pharyngeal Dysphagia in Patients with Parkinson's Disease. J Parkinsons Dis. 2020;10(4):1727-1735. doi: 10.3233/JPD-202081. PMID: 32773397.

Coates C, Bakheit AMO. Dysphagia in parkinson’s disease. Eur Neurol [Internet]. 1997 Jan 1 [cited 2020 Apr 18];38(1):49–52. Available from: <http://www.ncbi.nlm.nih.gov/pubmed/9252799>

Cosentino G, Tassorelli C, Prunetti P, Todisco M, De Icco R, Avenali M, Minafra B, Zangaglia R, Valentino F, Pacchetti C, Bertino G, Mauramati S, Fresia M, Alfonsi E. Reproducibility and reaction time of swallowing as markers of dysphagia in parkinsonian syndromes. Clin Neurophysiol. 2020 Sep;131(9):2200-2208.

Curtis JA, Molfenter S, Troche MS. Predictors of Residue and Airway Invasion in Parkinson's Disease. Dysphagia. 2020 Apr;35(2):220-230. doi: 10.1007/s00455-019-10014-z. Epub 2019 Apr 27. PMID: 31028481.

Curtis JA, Molfenter SM, Troche MS. Pharyngeal Area Changes in Parkinson's Disease and Its Effect on Swallowing Safety, Efficiency, and Kinematics. Dysphagia. 2020 Apr;35(2):389-398.

Curtis JA, Troche MS. Handheld Cough Testing: A Novel Tool for Cough Assessment and Dysphagia Screening. Dysphagia. 2020 Dec;35(6):993-1000. doi: 10.1007/s00455-020-10097-z. Epub 2020 Feb 24. PMID: 32095899.

Ding X, Gao J, Xie C, Xiong B, Wu S, Cen Z, et al. Prevalence and clinical correlation of dysphagia in Parkinson disease: a study on Chinese patients. Eur J Clin Nutr [Internet]. 2018 Jan 1 [cited 2020 Apr 20];72(1):82–6. Available from: <http://www.ncbi.nlm.nih.gov/pubmed/28699630>

Dumican M, Watts C. Predicting Airway Invasion Using Screening Tools and Laryngeal Kinematics in People with Parkinson's Disease: A Pilot Study. J Parkinsons Dis. 2020;10(3):1153-1160. doi: 10.3233/JPD-202044. PMID: 32538868; PMCID: PMC7458512.

Ellerston JK, Heller AC, Houtz DR, Kendall KA. Quantitative measures of swallowing deficits in patients with Parkinson s disease. Ann Otol Rhinol Laryngol. 2016;125(5):385–92.

Ertekin C, Tarlaci S, Aydogdu I, et al. Electrophysiological evaluation of pharyngeal phase of swallowing in patients with Parkinson's disease. Mov Disord. 2002;17(5):942‐949. doi:10.1002/mds.10240

Frank U, Radtke J, Nienstedt JC, Pötter-Nerger M, Schönwald B, Buhmann C, Gerloff C, Niessen A, Flügel T, Koseki JC, Pflug C. Dysphagia Screening in Parkinson's Disease. A diagnostic accuracy cross-sectional study investigating the applicability of the Gugging Swallowing Screen (GUSS). Neurogastroenterol Motil. 2020 Nov 20:e14034. doi: 10.1111/nmo.14034. Epub ahead of print. PMID: 33217102.

Fuh JL, Lee RC, Wang SJ, Lin CH, Wang PN, Chiang JH, et al. Swallowing difficulty in Parkinson’s disease. Clin Neurol Neurosurg. 1997;99(2):106–12.

Gaeckle M, Domahs F, Kartmann A, Tomandl B, Frank U. Predictors of Penetration-Aspiration in Parkinson’s Disease Patients With Dysphagia: A Retrospective Analysis. Ann Otol Rhinol Laryngol. 2019 Aug 1;128(8):728–35.

Golabbakhsh M, Rajaei A, Derakhshan M, Sadri S, Taheri M, Adibi P. Automated acoustic analysis in detection of spontaneous swallows in Parkinson's disease. Dysphagia. 2014;29(5):572‐577. doi:10.1007/s00455-014-9547-4

Hammer MJ, Murphy CA, Abrams TM. Airway somatosensory deficits and dysphagia in parkinson’s disease. J Parkinsons Dis. 2013;3(1):39–44.

Hartelius L, Svensson P. Speech and Swallowing Symptoms Associated with Parkinson’s Disease and Multiple Sclerosis: A Survey. Folia Phoniatr Logop [online serial]. 1994;46:9–17. Accessed at: <https://www.karger.com/Article/FullText/266286>.

Hegland KW, Okun MS, Troche MS. Sequential voluntary cough and aspiration or aspiration risk in Parkinson’s disease. Lung. Springer New York LLC; 2014;192:601–608.

Hegland KW, Troche MS, Brandimore A, Okun MS, Davenport PW. Comparison of Two Methods for Inducing Reflex Cough in Patients With Parkinson’s Disease, With and Without Dysphagia. Dysphagia. Springer US; 2016;31:66–73.

Johnston BT, Castell JA, Stumacher S, Colcher A, Gideon RM, Li Q, et al. Comparison of swallowing function in Parkinson’s disease and progressive supranuclear palsy. Mov Disord [Internet]. 1997 May [cited 2020 Apr 20];12(3):322–7. Available from: http://www.ncbi.nlm.nih.gov/pubmed/9159726

Jones CA, Ciucci MR. Multimodal Swallowing Evaluation with High-Resolution Manometry Reveals Subtle Swallowing Changes in Early and Mid-Stage Parkinson Disease. J Parkinsons Dis [Internet]. 2016 Mar 30 [cited 2020 Apr 18];6(1):197–208. Available from: <http://www.ncbi.nlm.nih.gov/pubmed/26891176>

Jones CA, Hoffman MR, Lin L, Abdelhalim S, Jiang JJ, McCulloch TM. Identification of swallowing disorders in early and mid-stage Parkinson’s disease using pattern recognition of pharyngeal high-resolution manometry data. Neurogastroenterol Motil [online serial]. 2018;30:e13236. Accessed at: http://doi.wiley.com/10.1111/nmo.13236.

Kalf JG, Borm GF, De Swart BJ, Bloem BR, Zwarts MJ, Munneke M. Reproducibility and validity of patient-rated assessment of speech, swallowing, and saliva control in parkinson’s disease. Arch Phys Med Rehabil. 2011;92(7):1152–8.

Kanna Sv, Bhanu K. A simple bedside test to assess the swallowing dysfunction in Parkinson′s disease. Ann Indian Acad Neurol [online serial]. Medknow Publications; 2014;17:62. Accessed at: <http://www.annalsofian.org/text.asp?2014/17/1/62/128556>.

Kim J, Watts CR. A comparison of swallow-related submandibular contraction amplitude and duration in people with Parkinson's disease and healthy controls. Int J Speech Lang Pathol. 2020 May 28:1-8.

Kooi AH, Boo JP, Ng SY, Acharyya S, Goh KH, Tay KY, Au WL, Tan LC. The Modified Barium Swallow Impairment Profile as a Predictor of Clinical Outcomes of Admission for Pneumonia or Choking in Dysphagic Patients with Parkinson's Disease. Dysphagia. 2019 Dec;34(6):896-903.

Lam K, Kwai Yi Lam F, Kwong Lau K, et al. Simple clinical tests may predict severe oropharyngeal dysphagia in Parkinson’s disease. Mov Disord [online serial]. 2007;22:640–644. Accessed at: http://doi.wiley.com/10.1002/mds.21362.

Lee KD, Koo JH, Song SH, Jo KD, Lee MK, Jang W. Central cholinergic dysfunction could be associated with oropharyngeal dysphagia in early Parkinson’s disease. J Neural Transm [Internet]. 2015 Nov 1 [cited 2020 Apr 20];122(11):1553–61. Available from: http://www.ncbi.nlm.nih.gov/pubmed/26199040

Lee WH, Lim MH, Nam HS, Kim YJ, Seo HG, Bang MS, et al. Differential kinematic features of the hyoid bone during swallowing in patients with Parkinson’s disease. J Electromyogr Kinesiol. 2019 Aug 1;47:57–64.

Mamolar Andrés S, Santamarina Rabanal ML, Granda Membiela CM, Fernández Gutiérrez MJ, Sirgo Rodríguez P, Álvarez Marcos C. Swallowing Disorders in Parkinson’s Disease. Acta Otorrinolaringol (English Ed [online serial]. Elsevier España, S.L.U. and Sociedad Española de Otorrinolaringología y Cirugía de Cabeza y Cuello; 2017;68:15–22. Accessed at: http://dx.doi.org/10.1016/j.otoeng.2017.01.003.

Manor Y, Giladi N, Cohen A, Fliss DM, Cohen JT. Validation of a swallowing disturbance questionnaire for detecting dysphagia in patients with Parkinson’s disease. Mov Disord [online serial]. 2007;22:1917–1921. Accessed at: http://doi.wiley.com/10.1002/mds.21625.

Mari F, Matei M, Ceravolo MG, Pisani A, Montesi A, Provinciali L. Predictive value of clinical indices in detecting aspiration in patients with neurological disorders. J Neurol Neurosurg Psychiatry. 1997;63:456–460.

Minagi Y, Ono T, Hori K, et al. Relationships between dysphagia and tongue pressure during swallowing in Parkinson’s disease patients. J Oral Rehabil. 2018;45:459–466.

Monte FS, da Silva-Júnior FP, Braga-Neto P, Nobre e Souza MA, de Bruin VMS. Swallowing abnormalities and dyskinesia in Parkinson’s disease. Mov Disord [Internet]. 2005 Apr [cited 2020 Apr 20];20(4):457–62. Available from: http://www.ncbi.nlm.nih.gov/pubmed/15625689

Monteiro L, Souza-Machado A, Pinho P, Sampaio M, Nóbrega AC, Melo A. Swallowing impairment and pulmonary dysfunction in Parkinson’s disease: The silent threats. J Neurol Sci. 2014 Apr 15;339(1–2):149–52.

Moreau C, Devos D, Baille G, Delval A, Tard C, Perez T, et al. Are Upper-Body Axial Symptoms a Feature of Early Parkinson’s Disease? PLoS One [Internet]. 2016 Sep 1 [cited 2020 Apr 20];11(9):e0162904. Available from: http://www.ncbi.nlm.nih.gov/pubmed/27654040

Nagaya M, Kachi T, Yamada T, Igata A. Videofluorographic study of swallowing in Parkinson’s disease. Dysphagia [Internet]. 1998 [cited 2020 Apr 18];13(2):95–100. Available from: <http://www.ncbi.nlm.nih.gov/pubmed/9513304>

Nienstedt JC, Bihler M, Niessen A, et al. Predictive clinical factors for penetration and aspiration in Parkinson’s disease. Neurogastroenterol Motil. 2019;31:1–9.

Nienstedt JC, Buhmann C, Bihler M, et al. Drooling is no early sign of dysphagia in Parkinson′s disease. Neurogastroenterol Motil. 2018;30:1–6.

Nóbrega AC, Rodrigues B, Torres AC, Scarpel RDA, Neves CA, Melo A. Is drooling secondary to a swallowing disorder in patients with Parkinson’s disease? Park Relat Disord. 2008;14(3):243–5.

Oh EH, Seo JS, Kang HJ. Assessment of Oropharyngeal Dysphagia in Patients With Parkinson Disease: Use of Ultrasonography. Ann Rehabil Med. 2016 Apr;40(2):190-6. doi: 10.5535/arm.2016.40.2.190. Epub 2016 Apr 25.

Perez-Lloret S, Nègre-Pagès L, Ojero-Senard A, et al. Oro-buccal symptoms (dysphagia, dysarthria, and sialorrhea) in patients with Parkinson’s disease: Preliminary analysis from the French COPARK cohort. Eur J Neurol. 2012;19:28–37.

Pflug C, Bihler M, Emich K, et al. Critical Dysphagia is Common in Parkinson Disease and Occurs Even in Early Stages: A Prospective Cohort Study. Dysphagia. 2018;33:41–50.

Pflug C, Niessen A, Buhmann C, Bihler M. Swallowing speed is no adequate predictor of aspiration in Parkinson's disease. Neurogastroenterol Motil. 2019 Dec;31(12):e13713. doi: 10.1111/nmo.13713. Epub 2019 Aug 29. PMID: 31464354.

Pitts LL, Morales S, Stierwalt JAG. Lingual Pressure as a Clinical Indicator of Swallowing Function in Parkinson’s Disease. J Speech Lang Hear Res. American Speech-Language-Hearing Association; 2018;61:257–265.

Pitts T, Troche M, Mann G, Rosenbek J, Okun MS, Sapienza C. Using voluntary cough to detect penetration and aspiration during oropharyngeal swallowing in patients with Parkinson disease. Chest [online serial]. The American College of Chest Physicians; 2010;138:1426–1431. Accessed at: http://dx.doi.org/10.1378/chest.10-0342.

Potulska A, Friedman A, Królicki L, Spychala A. Swallowing disorders in Parkinson’s disease. Parkinsonism Relat Disord [online serial]. 2003;9:349–353. Accessed at: <https://linkinghub.elsevier.com/retrieve/pii/S1353802003000452>.

Rajaei A, Ashtari F, Azargoon SA, et al. The association between saliva control, silent saliva penetration, aspiration, and videofluoroscopic findings in Parkinson’s disease patients. Adv Biomed Res. 2015;4:108.

Rodrigues B, Nóbrega AC, Sampaio M, Argolo N, Melo A. Silent saliva aspiration in Parkinson’s disease. Mov Disord [Internet]. 2011 Jan [cited 2020 Apr 18];26(1):138–41. Available from: <http://www.ncbi.nlm.nih.gov/pubmed/21322025>

Sampaio M, Argolo N, Melo A, Nóbrega AC. Wet voice as a sign of penetration/aspiration in Parkinson’s disease: Does testing material matter? Dysphagia. 2014;29:610–615.

Schiffer BL, Kendall K. Changes in Timing of Swallow Events in Parkinson’s Disease. Ann Otol Rhinol Laryngol. 2019 Jan 1;128(1):22–7.

Schlickewei O, Nienstedt JC, Frank U, Fründt O, Pötter-Nerger M, Gerloff C, Buhmann C, Müller F, Lezius S, Koseki JC, Pflug C. The ability of the eating assessment tool-10 to detect penetration and aspiration in Parkinson's disease. Eur Arch Otorhinolaryngol. 2020 Sep 26. doi: 10.1007/s00405-020-06377-x. Epub ahead of print. PMID: 32978686.

Schröder JB, Marian T, Claus I, et al. Substance P Saliva Reduction Predicts Pharyngeal Dysphagia in Parkinson’s Disease. Front Neurol. 2019;10:1–5.

Silverman EP, Carnaby G, Singletary F, Hoffman-Ruddy B, Yeager J, Sapienza C. Measurement of voluntary cough production and airway protection in parkinson disease. Arch Phys Med Rehabil [online serial]. Elsevier Ltd; 2016;97:413–420. Accessed at: <http://dx.doi.org/10.1016/j.apmr.2015.10.098>.

Simons JA, Fietzek UM, Waldmann A, Warnecke T, Schuster T, Ceballos-Baumann AO. Development and validation of a new screening questionnaire for dysphagia in early stages of Parkinson’s disease. Parkinsonism Relat Disord [online serial]. 2014;20:992–998. Accessed at: https://linkinghub.elsevier.com/retrieve/pii/S1353802014002284.

Singer C, Weiner WJ, Sanchez-Ramos JR. Autonomic Dysfunction in Men with Parkinson’s Disease. Eur Neurol [online serial]. 1992;32:134–140. Accessed at: <https://www.karger.com/Article/FullText/116810>.

Stroudley J, Walsh M. Radiological assessment of dysphagia in Parkinson’s disease. Br J Radiol. 1991;64:890–893.

Su A, Gandhy R, Barlow C, Triadafilopoulos G. Clinical and manometric characteristics of patients with Parkinson’s disease and esophageal symptoms. Dis esophagus Off J Int Soc Dis Esophagus [Internet]. 2017 Apr 1 [cited 2020 Apr 18];30(4):1–6. Available from: http://www.ncbi.nlm.nih.gov/pubmed/28375482

Suttrup I, Suttrup J, Suntrup-Krueger S, Siemer M-L, Bauer J, Hamacher C, et al. Esophageal dysfunction in different stages of Parkinson’s disease. Neurogastroenterol Motil [Internet]. 2017 Jan 1 [cited 2020 Apr 18];29(1). Available from: http://www.ncbi.nlm.nih.gov/pubmed/27477636

Taira K, Fujiwara K, Fukuhara T, Koyama S, Morisaki T, Takeuchi H. Evaluation of the pharynx and upper esophageal sphincter motility using high-resolution pharyngeal manometry for Parkinson's disease. Clin Neurol Neurosurg. 2021 Feb;201:106447.

Troche MS, Brandimore AE, Foote KD, et al. Swallowing Outcomes Following Unilateral STN vs. GPi Surgery: A Retrospective Analysis. Dysphagia [online serial]. 2014;29:425–431. Accessed at: <http://link.springer.com/10.1007/s00455-014-9522-0>.

Troche MS, Schumann B, Brandimore AE, Okun MS, Hegland KW. Reflex Cough and Disease Duration as Predictors of Swallowing Dysfunction in Parkinson’s Disease. Dysphagia. Springer US; 2016;31:757–764.

Umemoto G, Fujioka S, Arahata H, Kawazoe M, Sakae N, Sasagasako N, Furuya H, Tsuboi Y. Relationship between tongue pressure and functional oral intake scale diet type in patients with neurological and neuromuscular disorders. Clin Neurol Neurosurg. 2020 Nov;198:106196. doi: 10.1016/j.clineuro.2020.106196. Epub 2020 Sep 13. PMID: 32980799.

Vogel AP, Rommel N, Sauer C, et al. Clinical assessment of dysphagia in neurodegeneration (CADN): development, validity and reliability of a bedside tool for dysphagia assessment. J Neurol. Springer Berlin Heidelberg; 2017;264:1107–1117.

Volonte’ MA, Porta M, Comi G. Clinical assessment of dysphagia in early phases of Parkinson’s disease. Neurol Sci [online serial]. 2002;23:s121–s122. Accessed at: <http://link.springer.com/10.1007/s100720200099>.

Wakasugi Y, Yamamoto T, Oda C, Murata M, Tohara H, Minakuchi S. Effect of an impaired oral stage on swallowing in patients with Parkinson’s disease. J Oral Rehabil. 2017 Oct 1;44(10):756–62.

Walker RW, Dunn JR, Gray WK. Self-reported dysphagia and its correlates within a prevalent population of people with Parkinson’s disease. Dysphagia. 2011;26:92–96.

Wang C-M, Shieh W-Y, Weng Y-H, Hsu Y-H, Wu Y-R. Non-invasive assessment determine the swallowing and respiration dysfunction in early Parkinson’s disease. Parkinsonism Relat Disord [Internet]. 2017 Sep 1 [cited 2020 Apr 20];42:22–7. Available from: http://www.ncbi.nlm.nih.gov/pubmed/28606443

Ws Coriolano MDG, R Belo L, Carneiro D, G Asano A, Al Oliveira PJ, Da Silva DM, et al. Swallowing in patients with Parkinson’s disease: A surface electromyography study. Dysphagia. 2012 Dec;27(4):550–5.

Yiu Y, Curtis JA, Perry SE, Troche MS. Relationship of vocal fold atrophy to swallowing safety and cough function in Parkinson's disease. Laryngoscope. 2020 Feb;130(2):303-308.

Zilli Canedo Silva M, Carol Fritzen N, de Oliveira M, et al. Protein intake, nitrogen balance and nutritional status in patients with Parkinson's disease; time for a change?. Nutr Hosp. 2015;31(6):2764‐2770. Published 2015 Jun 1. doi:10.3305/nh.2015.31.6.8938

**QUALITY OF LIFE AND PROGNOSIS**

Akbar U, Dham B, He Y, Hack N, Wu S, Troche M, et al. Incidence and mortality trends of aspiration pneumonia in Parkinson’s disease in the United States, 1979-2010. Parkinsonism Relat Disord [Internet]. 2015 Sep 1 [cited 2020 Apr 20];21(9):1082–6. Available from: http://www.ncbi.nlm.nih.gov/pubmed/26154915

Auyeung M, Tsoi TH, Mok V, Cheung CM, Lee CN, Li R, et al. Ten year survival and outcomes in a prospective cohort of new onset Chinese Parkinson’s disease patients. J Neurol Neurosurg Psychiatry [Internet]. 2012 Jun [cited 2020 Apr 20];83(6):607–11. Available from: http://www.ncbi.nlm.nih.gov/pubmed/22362919

Barichella M, Cereda E, Madio C, Iorio L, Pusani C, Cancello R, et al. Nutritional risk and gastrointestinal dysautonomia symptoms in Parkinson’s disease outpatients hospitalised on a scheduled basis. Br J Nutr [Internet]. 2013 Jul 28 [cited 2020 Apr 20];110(2):347–53. Available from: <http://www.ncbi.nlm.nih.gov/pubmed/23228187>

Carneiro D, das Graças Wanderley de Sales Coriolano M, Belo LR, de Marcos Rabelo AR, Asano AG, Lins OG. Quality of life related to swallowing in Parkinson’s disease. Dysphagia. 2014;29:578–582.

Cereda E, Cilia R, Klersy C, Canesi M, Zecchinelli AL, Mariani CB, et al. Swallowing disturbances in Parkinson’s disease: a multivariate analysis of contributing factors. Parkinsonism Relat Disord [Internet]. 2014 Dec 1 [cited 2020 Apr 20];20(12):1382–7. Available from: http://www.ncbi.nlm.nih.gov/pubmed/25456827

Cilia R, Cereda E, Klersy C, Canesi M, Zecchinelli AL, Mariani CB, et al. Parkinson’s disease beyond 20 years. J Neurol Neurosurg Psychiatry. 2015 Aug 1;86(8):849–55.

Coelho M, Marti MJ, Tolosa E, et al. Late-stage Parkinson’s disease: The Barcelona and Lisbon cohort. J Neurol. 2010;257:1524–1532.

Curtis JA, Molfenter S, Troche MS. Predictors of Residue and Airway Invasion in Parkinson's Disease. Dysphagia. 2020 Apr;35(2):220-230. doi: 10.1007/s00455-019-10014-z. Epub 2019 Apr 27. PMID: 31028481.

Fabbri M, Coelho M, Abreu D, Guedes LC, Rosa MM, Godinho C, et al. Dysphagia predicts poor outcome in late-stage Parkinson’s disease. Parkinsonism Relat Disord [Internet]. 2019 Jul 1 [cited 2020 Apr 20];64:73–81. Available from: http://www.ncbi.nlm.nih.gov/pubmed/30902528

Goh KH, Acharyya S, Ng SYE, Boo JPL, Kooi AHJ, Ng HL, et al. Risk and prognostic factors for pneumonia and choking amongst Parkinson’s disease patients with dysphagia. Park Relat Disord. 2016 Aug 1;29:30–4.

Han M, Ohnishi H, Nonaka M, et al. Relationship between dysphagia and depressive states in patients with Parkinson’s disease. Park Relat Disord [online serial]. Elsevier Ltd; 2011;17:437–439. Accessed at: http://dx.doi.org/10.1016/j.parkreldis.2011.03.006.

Hussain J, Allgar V, Oliver D. Palliative care triggers in progressive neurodegenerative conditions: An evaluation using a multi-centre retrospective case record review and principal component analysis. Palliat Med [Internet]. 2018 Apr 1 [cited 2020 Apr 20];32(4):716–25. Available from: http://www.ncbi.nlm.nih.gov/pubmed/29400144

Lee JH, Lee KW, Kim SB, Lee SJ, Chun SM, Jung SM. The Functional Dysphagia Scale Is a Useful Tool for Predicting Aspiration Pneumonia in Patients With Parkinson Disease. Ann Rehabil Med [Internet]. 2016 Jun [cited 2020 Apr 20];40(3):440–6. Available from: <http://www.ncbi.nlm.nih.gov/pubmed/27446780>

Leow LP, Huckabee M-L, Anderson T, Beckert L. The Impact of Dysphagia on Quality of Life in Ageing and Parkinson’s Disease as Measured by the Swallowing Quality of Life (SWAL-QOL) Questionnaire. Dysphagia [online serial]. 2010;25:216–220. Accessed at: http://link.springer.com/10.1007/s00455-009-9245-9.

Lim A, Leow L, Huckabee M-L, Frampton C, Anderson T. A pilot study of respiration and swallowing integration in Parkinson’s disease: “on” and “off” levodopa. Dysphagia [Internet]. 2008 Mar [cited 2020 Apr 18];23(1):76–81. Available from: http://www.ncbi.nlm.nih.gov/pubmed/17602261

Lo RY, Tanner CM, Albers KB, Leimpeter AD, Fross RD, Bernstein AL, et al. Clinical features in early Parkinson disease and survival. Arch Neurol [Internet]. 2009 Nov [cited 2020 Apr 20];66(11):1353–8. Available from: <http://www.ncbi.nlm.nih.gov/pubmed/19901166>

Lorefält B, Granérus AK, Unosson M. Avoidance of solid food in weight losing older patients with Parkinson’s disease. J Clin Nurs. Epub 2006.

Malmgren A, Hede GW, Karlström B, Cederholm T, Lundquist P, Wirén M, et al. Indications for percutaneous endoscopic gastrostomy and survival in old adults. Food Nutr Res [Internet]. 2011 [cited 2020 Apr 20];55. Available from: <http://www.ncbi.nlm.nih.gov/pubmed/21799666>

Manor Y, Balas M, Giladi N, Mootanah R, Cohen JT. Anxiety, depression and swallowing disorders in patients with Parkinson’s disease. Park Relat Disord. Epub 2009.

Martinez-Ramirez D, Almeida L, Giugni JC, et al. Rate of aspiration pneumonia in hospitalized Parkinson's disease patients: a cross-sectional study. BMC Neurol. 2015;15:104. Published 2015 Jul 5. doi:10.1186/s12883-015-0362-9

Merola A, Zibetti M, Angrisano S, et al. Parkinson's disease progression at 30 years: a study of subthalamic deep brain-stimulated patients. Brain. 2011;134(Pt 7):2074‐2084. doi:10.1093/brain/awr121

Miller N, Allcock L, Hildreth AJ, Jones D, Noble E, Burn DJ. Swallowing problems in Parkinson disease: frequency and clinical correlates. J Neurol Neurosurg Psychiatry [online serial]. 2009;80:1047–1049. Accessed at: <http://jnnp.bmj.com/cgi/doi/10.1136/jnnp.2008.157701>.

Miller N, Noble E, Jones D, Burn D. Hard to swallow: dysphagia in Parkinson’s disease. AgeAgeing [online serial]. 2006;35:614–618. Accessed at: <http://academic.oup.com/ageing/article/35/6/614/14909/Hard-to-swallow-dysphagia-in> Parkinsons-disease.

Miyazaki Y, Arakawa M, Kizu J. Introduction of simple swallowing ability test for prevention of aspiration pneumonia in the elderly and investigation of factors of swallowing disorders. Yakugaku Zasshi [Internet]. 2002 Jan [cited 2020 Apr 18];122(1):97–105. Available from: http://www.ncbi.nlm.nih.gov/pubmed/11828754

Müller J, Wenning GK, Verny M, McKee A, Chaudhuri KR, Jellinger K, et al. Progression of dysarthria and dysphagia in postmortem-confirmed parkinsonian disorders. Arch Neurol [Internet]. 2001 Feb 1 [cited 2019 Aug 22];58(2):259–64. Available from: <http://archneur.jamanetwork.com/article.aspx?doi=10.1001/archneur.58.2.259>

Ou R, Guo X, Wei Q, et al. Prevalence and clinical correlates of drooling in Parkinson disease: A study on 518 Chinese patients. Park Relat Disord [online serial]. Elsevier; 2015;21:211–215. Accessed at: http://dx.doi.org/10.1016/j.parkreldis.2014.12.004.

Pitts T, Bolser D, Rosenbek J, Troche M, Sapienza C. Voluntary cough production and swallow dysfunction in Parkinson's disease. Dysphagia. 2008;23(3):297‐301. doi:10.1007/s00455-007-9144

Plowman-Prine EK, Sapienza CM, Okun MS, et al. The relationship between quality of life and swallowing in Parkinson’s disease. Mov Disord. Epub 2009.

Robbins J, Gensler G, Hind J, Logemann JA, Lindblad AS, Brandt D, et al. Comparison of 2 interventions for liquid aspiration on pneumonia incidence: A randomized trial. Ann Intern Med. 2008 Apr 1;148(7):509–18.

Silbergleit AK, Lewitt P, Junn F, et al. Comparison of dysphagia before and after deep brain stimulation in Parkinson’s disease. Mov Disord. 2012;27:1763–1768.

Storch A, Schneider CB, Wolz M, et al. Nonmotor fluctuations in Parkinson disease: Severity and correlation with motor complications. Neurology. 2013;80:800–809.

Tomita S, Oeda T, Umemura A, Kohsaka M, Park K, Yamamoto K, et al. Video-fluoroscopic swallowing study scale for predicting aspiration pneumonia in Parkinson’s disease. PLoS One [Internet]. 2018 Jun 1 [cited 2020 Apr 18];13(6):e0197608. Available from: <http://www.ncbi.nlm.nih.gov/pubmed/29874285>

Van Hooren MRA, Baijens LWJ, Vos R, et al. Voice- and swallow-related quality of life in idiopathic Parkinson’s disease. Laryngoscope. 2016;126:408–414.

Van Hooren MRA, Vos R, Florie MGMH, Pilz W, Kremer B, Baijens LWJ. Swallowing Assessment in Parkinson's Disease: Patient and Investigator Reported Outcome Measures are not Aligned. Dysphagia. 2020 Oct 31. doi: 10.1007/s00455-020-10201-3. Epub ahead of print. PMID: 33130951.

Wang CM, Tsai TT, Wang SH, Wu YR. Does the M.D. Anderson Dysphagia Inventory correlate with dysphagia-limit and the Unified Parkinson Disease Rating Scale in early-stage Parkinson's disease? J Formos Med Assoc. 2020 Jan;119(1 Pt 2):247-253. doi: 10.1016/j.jfma.2019.05.005. Epub 2019 May 24. PMID: 31133522.
